# Consensus-based framework for evaluating data modernization initiatives: the case of cancer registration and electronic reporting

**DOI:** 10.1093/jamiaopen/ooad060

**Published:** 2023-08-23

**Authors:** Sujha Subramanian, Florence K L Tangka, Paran Pordell, Jenny Beizer, Reda Wilson, Sandra F Jones, Joseph D Rogers, Vicki B Benard, Lisa C Richardson

**Affiliations:** Implenomics, Dover, Delaware, USA; Division of Cancer Prevention and Control, Centers for Disease Control and Prevention, Atlanta, Georgia, USA; Division of Cancer Prevention and Control, Centers for Disease Control and Prevention, Atlanta, Georgia, USA; RTI International, Research Triangle Park, North Carolina, USA; Division of Cancer Prevention and Control, Centers for Disease Control and Prevention, Atlanta, Georgia, USA; Division of Cancer Prevention and Control, Centers for Disease Control and Prevention, Atlanta, Georgia, USA; Division of Cancer Prevention and Control, Centers for Disease Control and Prevention, Atlanta, Georgia, USA; Division of Cancer Prevention and Control, Centers for Disease Control and Prevention, Atlanta, Georgia, USA; Division of Cancer Prevention and Control, Centers for Disease Control and Prevention, Atlanta, Georgia, USA

**Keywords:** cancer surveillance, electronic reporting, data modernization initiative

## Abstract

As part of its data modernization initiative (DMI), the Centers for Disease Control and Prevention, Division of Cancer Prevention and Control is testing and implementing innovative solutions to improve cancer surveillance data quality and timeliness. We describe a consensus-based effort to create a framework to guide the evaluation of cancer surveillance modernization efforts by addressing specific context, processes, and costs related to cancer registration. We drew on prior theories, consulted with experts, and sought feedback from cancer registry staff. We developed the cancer surveillance systems, context, outcomes, and process evaluation (CS-SCOPE) framework to explain the ways in which cancer registry data quality, timeliness, and efficiency are impacted by external and internal contextual factors and interrelated process and content factors. The framework includes implementation measures to understand acceptability of process changes along with outcome measures to assess DMI initiation and ongoing sustainability. The framework’s components and structures can be tailored for use in other DMI evaluations.

## BACKGROUND

Over the past decade, national, state, and local public health agencies have launched initiatives to modernize healthcare data and drive better decision-making by removing silos and improving connectivity.^1^^,^^2^ Many agencies, including the Centers for Disease Control and Prevention (CDC), have launched multiyear data modernization initiatives (DMI).^3^ CDC’s initiative is modernizing core data and surveillance infrastructure across the federal and state public health landscape by updating technology and ensuring the right people, processes, and policies are available.^3^^,^^4^

As part of its DMI activities, CDC’s Division of Cancer Prevention and Control is developing and testing innovative solutions to improve cancer surveillance data quality and timeliness.^5–7^ Data from cancer registries serve a vital role in assessing cancer burden and improving health outcomes by providing details on cancer incidence along with mortality and survival data through linkages with state vital records.^8^^,^^9^ Each year, cancer registries identify, process, and submit approximately 1.7 million reportable cancer cases to the National Program of Cancer Registries (NPCR) as part of ongoing population-based cancer surveillance efforts in the United States.^10^ Tools and other software developed by NPCR have improved cancer surveillance procedures,^11^ but challenges remain. Manual processes are often needed to finalize cases, and 24 months is usually required to report complete cancer surveillance data. Current modernization efforts are focused on increasing automation in transferring and processing data, with the overall goal of providing timely data for decision-making.^5^^,^^7^

To modernize cancer data collection and reporting, NPCR is developing and testing a cancer surveillance cloud-based computing platform (CS-CBCP).^5^ The goal of CS-CBCP is to offer an infrastructure that will allow cancer registries to engage in real-time cancer incidence case collection. This will be facilitated through centralized repositories for source data; increased automation and use of natural language processing; matching algorithms for tumors and patients; and rapid processes for reporting data. These anticipated improvements in timeliness and data quality, specifically accuracy and completeness (which will require review of multiple data sources), can enhance optimal implementation of population-level cancer prevention, along with better care delivery for individuals diagnosed with cancer.

CDC places a high emphasis on effective program evaluation, through the use of frameworks, logic models, and measurement indicators, as this offers a systematic way to identify optimal approaches to improve and account for public health actions.^12^ In this article, we present a framework for the comprehensive evaluation of cancer surveillance modernization efforts that expands on CDC’s DMI monitoring and evaluation and efforts to address the specific context, processes, and cost related to cancer registration.^13^ We developed the cancer surveillance systems, context, outcomes, and process evaluation (CS-SCOPE) framework to explain the ways in which cancer registry data quality and efficiency are impacted by multiple interlinked elements. Registry operations are affected by external and internal contextual factors along with the procedures and support systems that are in place to receive, process, and report data. The overall premise of this framework is based on the acknowledgment that NPCR’s DMI initiatives have to consider the adaptations and support strategies needed to increase adoption of electronic reporting. It will be important for registries to plan for initial investments along with process changes, which could result in efficiencies and cost savings in the future. We anticipate that these changes will occur over several iterations as challenges are identified and solution are tested to facilitate sustainment of changes. The proposed framework can be used to conduct systematic evaluations among cancer registries with varied ecosystems to ensure successful implementation, optimization, and sustainment of modernization efforts.

## METHODS TO DEVELOP CS-SCOPE FRAMEWORK

**Figure 1.**
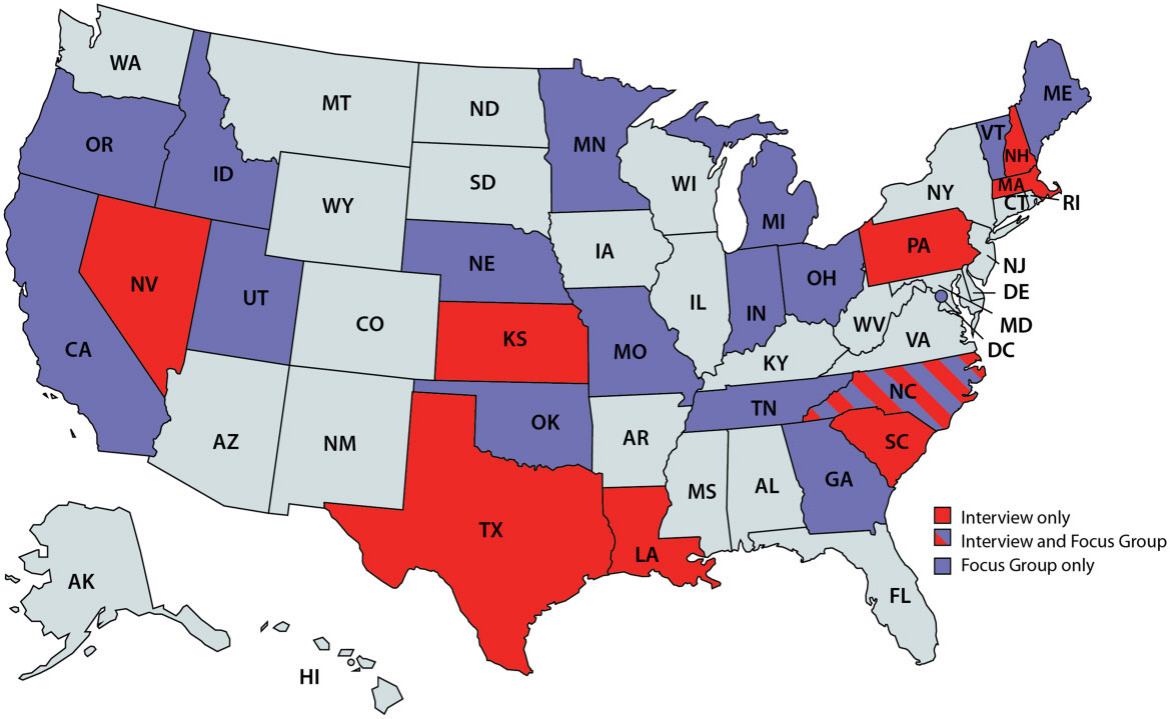
Registries participating in interviews or focus groups. *Note*: States in light blue were not included in the study.

Our goal was to develop a consensus-based, theory-driven framework to guide evaluation of cancer surveillance modernization efforts. We began with the theoretical constructs proposed by Pettigrew^14^ to guide change management and transformation, which include the interactions between broad categories related to context, process, and content. The team reviewed multiple other change management models, including Lewin’s 3-step model, Kotter’s change management theory, McKinsey’s 7-S framework, and Bridges transition model.^15^ There was unanimous consensus that the Pettigrew framework was the most appropriate for the registry contextual features and DMI focus. The context includes the internal and external factors that can impact organizational decision-making and implementation.^16^ Process consists of the underlying procedural steps that are the drivers of the transformation. Content refers to the specific structures that can undergo changes. As a first step, we developed a potential set of constructs for each of the broad categories based on prior cancer registry evaluations and lessons from informatics implementation reviews.^17–20^ Second, we engaged CDC and state level experts in cancer registry operations to review and provide feedback on the initial CS-SCOPE framework constructs. These experts also reviewed interviews and focus group guides developed to further explore these constructs. Third, we interviewed 9 cancer registry directors and 9 data managers in early 2019, using the detailed interview guide and we then conducted 2 focus groups with registry directors and data managers to seek additional feedback on the findings from the interviews. The target sample size for the interviews and the focus groups was about 9 respondents based on the numbers required to reach saturation in our previous research with CDC programs and confirmed by others.^21–23^Table 1 provides details on the constructs and themes explored; Figure 1 presents the registries that participated. Registries were selected using a purposive sampling approach to ensure broad representation across NPCR. The detailed findings from the interviews on adoption of electronic data reporting have been previously published,^24^ and in this article, we summarize key aspects related to contextual factors using findings from both the interviews and focus groups.

**Table 1.** Qualitative data collection participants

Approach and registry team member	Constructs and themes explored	Participants (*N*)
NPCR Interview Guide—Registry Directors	Registry overview; facilitators and barriers to electronic reporting^a^; costs of operations and sustainability	9
NPCR Interview Guide—Registry Data Managers	Data collection processes; software and systems support; facilitators and barriers to electronic reporting; data quality and sustainability	9
Virtual Focus Group Guide—Facilitators and Barriers	Data processing goals; minimum thresholds for electronic reporting; facilitators and barriers to electronic reporting; technical assistance feedback	9
Virtual Focus Group Guide—Process Modifications and Outcomes	Data quality goals; key process impacts of electronic reporting; quality improvement impact of electronic reporting; cost impacts of electronic reporting; future innovative approaches	8

a Electronic reporting can be defined as any method that does not require manual intervention or data entry.

Two researchers kept detailed notes of each interview and focus group to ensure complete and thorough representation of the responses. Both sets of notes were compiled into a single, complete version and 2 researchers independently reviewed the notes to identify key constructs. Given the overall small number of interviews and focus groups, no other software was used for the analysis. There was overall strong agreement between the 2 researchers, and we convened with the larger project team to review and finalize the emergent themes and constructs. We compiled these constructs under the broad categories of external factors, internal factors, process, and content. The study team also identified the impacts of external and internal factors on 4 aspects of registry operations and outcomes: (1) electronic reporting; (2) cost; (3) data quality (completeness and accuracy); and (4) timeliness. Based on these findings, we updated constructs included in the framework related to context both in terms of external factors and internal factors. Information received through the interviews and focus groups also informed the process and content components included in the framework. Last, based on feedback received, we incorporated updated outcomes metrics to evaluate impact along with implementation measures (eg, acceptability and feasibility of the DMI-related process changes) to assess the change management process in the CS-SCOPE framework. CDC Institutional Review Board review of this project was not required.

## FRAMEWORK FOR EVALUATING CANCER SURVEILLANCE DMI

**Figure 2.**
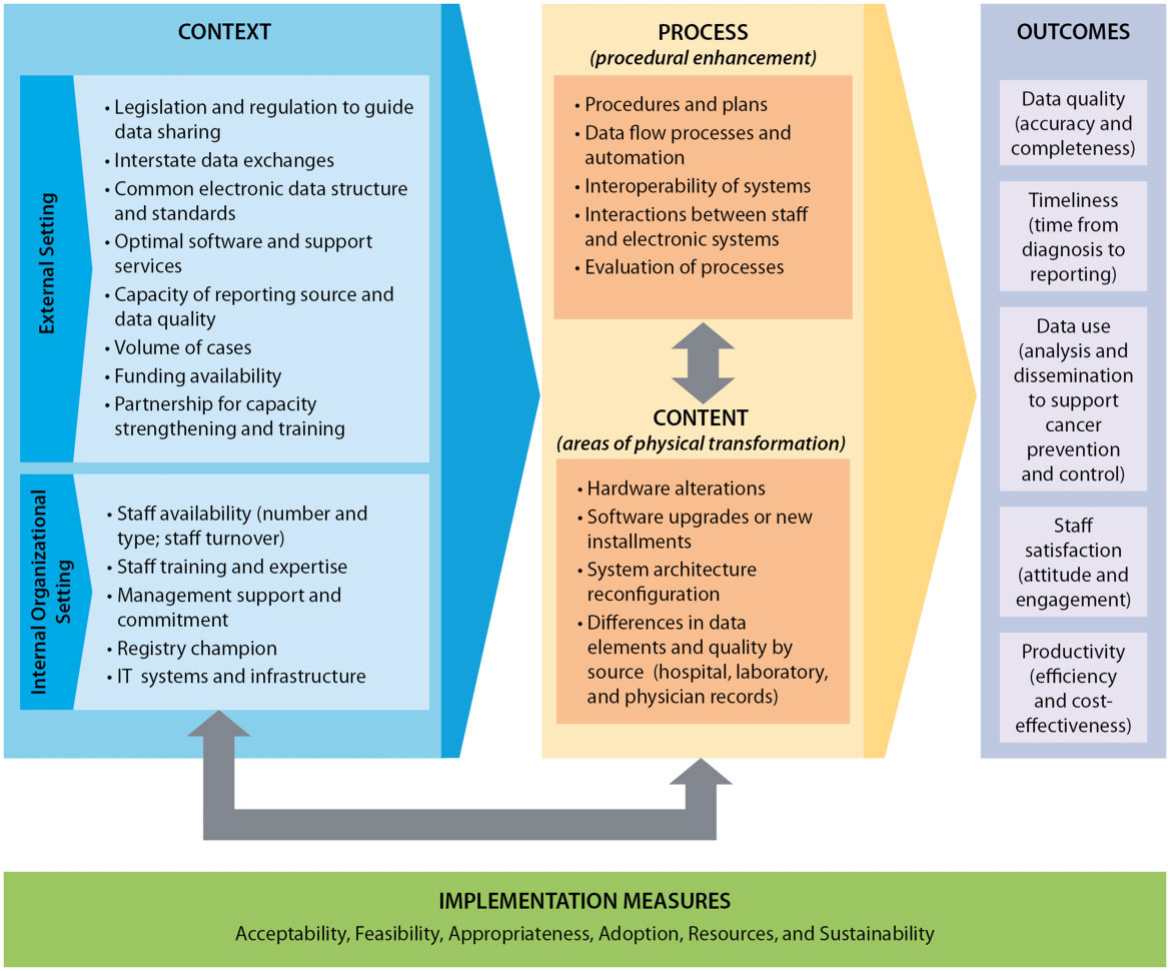
CS-SCOPE framework for systematic evaluation of data modernization initiatives. *Notes*: The definitions for the implementation measures are: Acceptability—The perspective of the registry team members impacted by the change as to whether the new approach is agreeable or palatable; Feasibility—The extent to which the new processes or systems can be successfully embedded within existing workflow to seamlessly integrate and continue operations; Appropriateness—The compatibility or relevance of the data modernization innovations to the overall goals of the implementing organizations; Adoption—The action of implementing the change or the “uptake” of the data modernization practice; Resources—The investment required for implementation related to the start-up phase and ongoing efforts. The changes implemented can impact the resources expended on specific activities, as well as the staffing mix and budget categories; Sustainability—The extent to which the changes implemented can be maintained and supported over time after completion of the initial planning and implementation phases.

The consensus-based CS-SCOPE framework for systematic evaluation of cancer surveillance data modernization efforts is presented in Figure 2. The central aspects of the CS-SCOPE framework are the procedural enhancements required for implementing data modernization (Process category) and areas of physical transformation related to software and hardware (Content category). These 2 categories are influenced by factors related to the external setting and internal organizational setting (Context category) and all these categories in turn impact the results of the DMIs (Outcomes category). Additionally, the framework incorporates measures to assess the implementation of the data modernization changes (Implementation Measures category) to provide information on why the implementation efforts are or are not achieving anticipated results.

Additional details on the key categories included in the framework are provided below.

- Context—These are external and internal factors that can impact the ability of cancer registries to plan, implement, and sustain procedures required to receive and process electronic data from multiple sources. External setting includes policies, procedures, regulations, and state resources for facilitating electronic data exchange along with financial and non-financial support. Internal organizational setting refers to the registry capacity, staff availability/capabilities, management commitment, and short- and long-term goals. Table 2 provides details on the contextual factors identified through the interviews and focus groups. We also present the potential impacts of the factors on electronic reporting, cost of operations, data quality, and timeliness.
- Process—This includes all factors related to the process of change, including implementation plans, data flow alterations, standard operating procedures, staff interaction with cloud-based systems, and evaluation of the implementation procedures. These action steps can lead to procedural enhancement in receiving and processing cancer registry data. A key aspect highlighted in our consultations is the importance of considering human and machine interactions as the activities conducted by each changes with new electronic reporting and automation. In some instances, the change leads to efficiencies and is a welcome enhancement but in other situations the staff face additional burden in terms of manual reviews to verify automated reports. Therefore, cloud-based enhancement should not exclude the staff interactions and activities that will be required.
- Content—This refers to the physical aspects that are specifically impacted when changes in data processing are implemented. In the case of electronic data transfers to cancer registries, these include the hardware and software used, as well as the data itself. The key areas of physical transformation identified through interviews and focus groups include the following: hardware alterations, software upgrades and new installments, system architecture reconfiguration, and differences in the quality of information available from data sources such as hospitals, laboratories, and physician clinics.
- Outcomes—The context, content, and process drive the outcomes in this framework, which include data quality (accuracy and completeness), timeliness, data use, staff satisfaction, and productivity. The aspects related to data quality and timeliness are key metrics included in NPCR quality standards reporting. Data use is also a standardized item collected in annual reporting to assess usability and impact of the cancer registry data. Staff satisfaction and productivity are new aspects identified through our consultation process as key essential outcomes that should be tracked and reported. Interview and focus group participants recommended these new constructs as they were deemed essential for evaluating the ability to sustain changes related to electronic reporting and automation.

**Table 2.** Summary of contextual factors related facilitators and barriers that affect registry outcomes

	Electronic reporting	Cost	Data quality	Timeliness	Feedback on facilitators and barriers
External factors					
Legislation and regulation	X			X	Facilitator: Most registries have legislation requiring electronic reporting
					Barrier: Legislation is not always enforced and does not always specify what qualifies as electronic
Interstate data exchange	X	X	X	X	Facilitator: Interstate data exchanges support completeness as registries obtain data on residents who seek care outside the registry’s geographic coverage area
Common electronic data structure and standards	X	X	X	X	Facilitator: Cancer registry-specific standards
Optimal software and support services	X	X	X	X	Barrier: Although data often come in electronically, registries may not have the software to support a fully automated electronic reporting system
Capacity and quality of reporting sources	X	X	X	X	Barrier: Smaller physician’s offices or laboratories often do not have the capacity, motivation, or resources to change to electronic reporting
Volume of cases received		X	X	X	Facilitator: Registries indicated that automation of processes was essential for efficiency
					Barrier: Increased volume often increases time spent on data processing and consolidation
Funding availability	X				Facilitator: Registries blend multiple funding sources to help achieve economies of scale and implement process changes
Partnerships for capacity-building and training	X	X	X	X	Facilitator: Partnerships, collaboration, and knowledge-sharing across registries and organizations support registries with key functions and process improvements
Internal factors					
Staff availability	X	X	X	X	Facilitator: Availability of staff with necessary expertise
					Barrier: Staff turnover and salary concerns
Staff training and expertise	X	X	X	X	Facilitator: Online training videos are efficient
					Barrier: Lack of technical skillset among staff
Management support and commitment	X		X	X	Facilitator: Guidance and long-term planning to support implementation and sustainability of processes
Registry champion	X		X	X	Facilitator: Leaders with knowledge to drive process improvements
IT systems/infrastructure	X	X	X	X	Facilitator: Systems and capabilities to receive and process electronic data

Implementation measures—These are metrics to monitor and manage implementation of changes initiated through DMI. These measures are based on implementation outcome measurements commonly used in the field of implementation science^25^ and can be assessed using both qualitative and quantitative approaches. They include aspects related to acceptability, feasibility, appropriateness, adoption, resources, and sustainability. For example, acceptability can be assessed by directly asking implementors in surveys or through interviews. Aspects related to resource use can be identified through financial analysis using existing accounting systems or through tailored activity-based cost assessment. Sustainability will have to be tracked over time to see which process and content changes are maintained over time and additionally, what type of modifications or adaptations may be required to sustain them. The arrows embedded in the CS-SCOPE framework (Figure 2) highlight the interlinkages between process, content, and inner organizational setting. A change to one of these 3 categories will impact the others. These interactions have to be recognized and accommodated in any data modernization efforts.

## APPLICATION TO CS-CBCP

**Figure 3.**
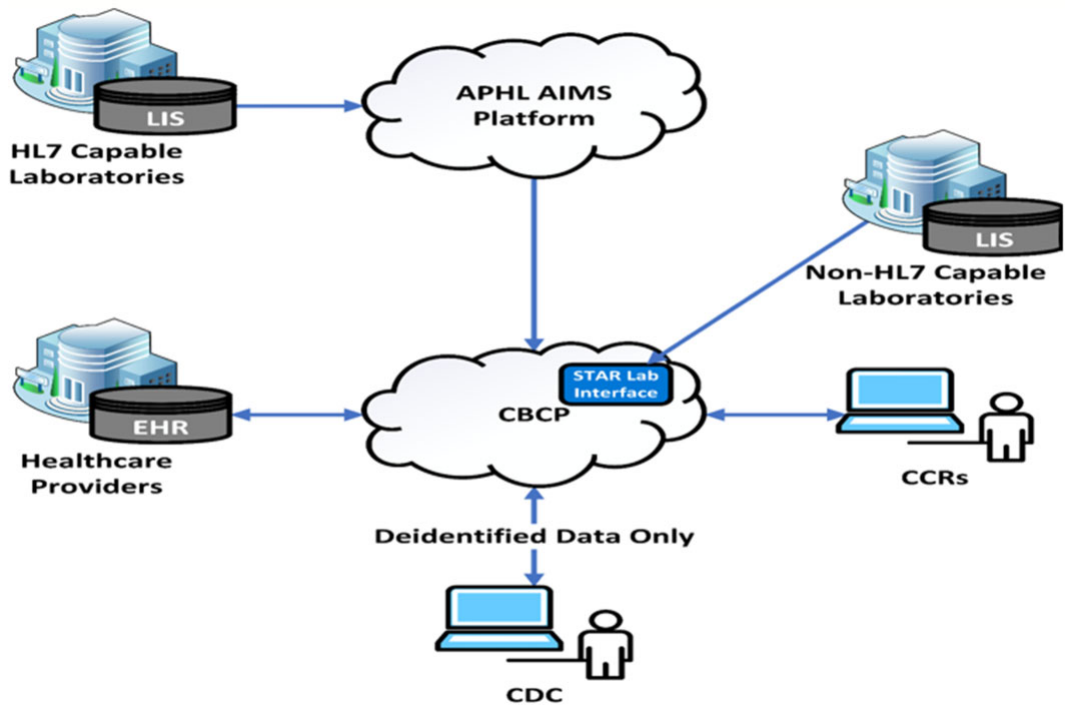
Cancer surveillance cloud-based computing platform (CS-CBCP) simplified data flow schematic. *Notes*: EHR: electronic health record; CCR: Central Cancer Registry; LIS: Laboratory Information System.

The CS-SCOPE framework presented in Figure 2 could offer guidance to trace the impacts of implementing CS-CBCP in terms of procedural modification, support structures, and outcomes. As shown in Figure 3, CS-CBCP will offer a centralized data repository and real-time data capture that will trigger process changes with newly created data flow procedures, increased automation, and enhanced interoperability for cancer registries. These process modifications will impact content features, such as the need for new software installations and will lead to changes in the type and quantity of data received from various reporting sources, including laboratories and healthcare professionals. Additionally, modifications may be required to the computing infrastructure. The CS-CBCP will also impact the internal organizational setting as staff will need to be trained or hired to support new processes. Thus, implementation of CS-CBCP will trigger changes in process, content, and internal organizational setting components of the model. Furthermore, the external contextual factors presented in Figure 2 could server as barriers or facilitators to implementing electronic data reporting and enhancing automation. Thus, the external setting factors are important determinants of implementation success. For example, rapid diffusion of CS-CBCP may require that state legislation and data-sharing laws be revised to align with CS-CBCP’s envisioned data flow pathways (eg, RI cancer registry legislation, 216-RICR-10-10-2, https://rules.sos.ri.gov/regulations/part/216-10-10-2). The key constructs included in each category of the framework can enable CDC staff and registries to plan, document, and track changes to monitor the implementation process and plan for successful adoption of CS-CBCP. Registries can formalize the evaluation of the implementation process by documenting changes (eg, software upgrades) and the investment required to make the modifications (eg, cost of installing software and conducting pilot testing) along with the outcomes (eg, improvement in data timeliness). All the information collected can then we used to conduct return on investment; for example, derive the cost of reducing reporting time by say 6 months. Furthermore, the registries can also assess which of the external and internal contextual factors were barriers or facilitators and which factors are essential to ensure sustainment.

Sustainability will depend on continued support for common standards and interoperability. Identifying and assessing impacts of these determinants will be essential to the ongoing successful implementation of CS-CBCP. The implementation measures identified in the framework will allow for monitoring of implementation outcomes, while the measures related to quality, timeliness, data use, and cost efficiency assessments will support evaluation of the outcome impacts. Resources required to initiate, implement, and sustain new processes and systems can be estimated to understand costs related to budget categories and registry activities. Lastly, productivity analysis and staff satisfaction can be used to evaluate the ability to sustain changes over time.

## DISCUSSION

The CS-SCOPE framework offers a consensus-driven model to evaluate DMI related to cancer surveillance. Data modernization approaches that target modifiable processes and systems can result in improvements in data quality, timeliness, and registry operations, but sustainability will likely require contextual factors that are in alignment with the changes implemented. The CS-SCOPE framework provides constructs related to the context, processes, and content areas to help identify and understand the impacts of these interrelated factors on the implementation of data improvement initiatives. The implementation measures and impact outcomes will enable CDC and registry partners to monitor and optimize strategies to support successful uptake. Potential strategies could include targeted technical assistance from experts, peer-to-peer implementation support and trainings for registry staff.

The methods applied to develop the framework have a few limitations that should be considered in applying the model in real-world practice. First, the registries selected for this study are a small subset of the central cancer registries; therefore, the findings may not be broadly generalizable. The registries were all based in the United States, and there may be implementation differences compared to other international registries. Second, the software and processes used, along with IT resources and support, differ among registries. So implementation of DMIs may vary substantially among registries. Finally, some of the external contextual factors may be related to policy decisions made by the state legislature, which may not be easily modifiable to support electronic reporting or automation.

As a next step, the NPCR evaluation team will develop definitions and measures to operationalize the CS-SCOPE framework using the NPCR performance measures as building blocks.^26^ We are working with 21 cancer registries to implement this framework and also formalize metrics in consultation with the registry staff. Many of the required data elements are already collected as part of ongoing monitoring and evaluation activities and include information collected via the Program Evaluation Instrument related to staff composition and software utilized.^27^ Additionally, data on registry data quality standards contain information on completeness, accuracy, and timeliness.^28^ Special studies conducted by CDC have collected resource use and activity-based cost data.^18^^,^^19^ This evaluation of the framework will occur prospectively over several annual periods and the team will report the results from this study in the future. New scales and data tools may be required to accurately capture implementation measures and outcomes, such as staff satisfaction. Pilot testing will be an important component of ongoing evaluations of electronic reporting procedures to assess accuracy and validity of any new measures that will need to be developed. We envision that the CS-SCOPE framework will offer a flexible model that can be adapted and updated as new information is learned through evaluation of cancer registration processes. Furthermore, this framework could be useful for other types of DMIs including those focused on chronic disease prevention and management that are implemented through enhancements to the electronic medical records. The categories of the framework have broad applicability to electronic reporting and automation but will require tailored metrics and measures to support each type of program area.

## Data Availability

Given the small sample sizes and qualitative nature of the data collection, transcripts will not be released as participant identity cannot be masked. The themes that emerged from the analysis of the interviews and focus groups have been reported in the article. We have also provided additional themes under Supplemental Materials. Data available from the Dryad Digital Repository: Doi: 10.5061/dryad.kh18932cd.
